# Spermatogonial stem cell sensitivity to capsaicin: An in vitro study

**DOI:** 10.1186/1477-7827-6-52

**Published:** 2008-11-14

**Authors:** Sefika C Mizrak, Bart M Gadella, Hatice Erdost, Aytekin Ozer, Ana MM van Pelt, Federica MF van Dissel-Emiliani

**Affiliations:** 1 Fertility Laboratory, Centre for Reproductive Medicine, Academic Medical Centre, Amsterdam, The Netherlands; 2 Department of Biochemistry and Cell Biology, Faculty of Veterinary Medicine, Utrecht University, Utrecht, The Netherlands; 3 Histology and Embryology Department, Faculty of Veterinary Medicine, Uludag University, Bursa, Turkey; 4 Department of Equine Sciences, Faculty of Veterinary Medicine, Utrecht University, Utrecht, The Netherlands

## Abstract

**Background:**

Conflicting reports have been published on the sensitivity of spermatogenesis to capsaicin (CAP), the pungent ingredient of hot chili peppers. Here, the effect of CAP on germ cell survival was investigated by using two testis germ cell lines as a model. As CAP is a potent agonist of the transient receptor potential vanilloid receptor 1 (TRPV1) and no information was available of its expression in germ cells, we also studied the presence of TRPV1 in the cultured cells and in germ cells in situ.

**Methods:**

The rat spermatogonial stem cell lines Gc-5spg and Gc-6spg were used to study the effects of different concentrations of CAP during 24 and 48 h. The response to CAP was first monitored by phase-contrast microscopy. As germ cells appear to undergo apoptosis in the presence of CAP, the activation of caspase 3 was studied using an anti activated caspase 3 antibody or by quantifying the amount of cells with DNA fragmentation using flow cytometry. Immunolocalization was done with an anti-TRPV1 antibody either with the use of confocal microscopy to follow live cell labeling (germ cells) or on Bouin fixed paraffin embedded testicular tissues. The expression of TRPV1 by the cell lines and germ cells was confirmed by Western blots.

**Results:**

Initial morphological observations indicated that CAP at concentrations ranging from 150 uM to 250 uM and after 24 and 48 h of exposure, had deleterious apoptotic-like effects on both cell lines: A large population of the CAP treated cell cultures showed signs of DNA fragmentation and caspase 3 activation. Quantification of the effect demonstrated a significant effect of CAP with doses of 150 uM in the Gc-5spg cell line and 200 uM in the Gc-6spg cell line, after 24 h of exposure. The effect was dose and time dependent in both cell lines. TRPV1, the receptor for CAP, was found to be expressed by the spermatogonial stem cells in vitro and also by premeiotic germ cells in situ.

**Conclusion:**

CAP adversely affects spermatogonial survival in vitro by inducing apoptosis to those cells and TRPV-1, a CAP receptor, may be involved in this effect as this receptor is expressed by mitotic germ cells.

## Background

Capsaicin (CAP 8-methyl-N-vanillyl-6-nonandamide) is a primary pungent and irritating principle present in hot peppers of the genus *Capsicum *which are widely and frequently consumed as food additive throughout the world [1]. Due to its ability to selectively excite and later desensitize nociceptor terminals, CAP has also been extensively used in the study of pain mechanisms. CAP formulations are now developed to treat a variety of diseases associated with neurogenic pain [2,3].

The widespread use of CAP as a food additive, topical analgesic or even self-defense product, necessitates an evaluation of its toxicity. Numerous studies have investigated the effect of CAP (extracts) on genotoxicity and mutagenicity on different cell types *in vitro *as well as *in vivo *[4-6]. However, the results are discordant, as some studies have showed that CAP has tumour-promoting potential [1,7] whereas others have suggested that this compound may be useful in the prevention or treatment of cancer due to its ability to inhibit the growth of transformed cells by inducing apoptosis [8-15].

Only a few and contradictory studies have investigated the effect of CAP on the reproductive system. *Nagabushan et al*. [16] found that CAP inhibits DNA synthesis in the testes of adult mice when injected intraperitoneally while *Muralidhara and Narasimhamurthy *[17] did not find any alteration in testicular weight and histology using similar doses. Remarkably, *Ozer et al*. [18] showed that CAP stimulates spermatogenic cell proliferation in developing roosters. Additionally these authors demonstrated that CAP accelerates the development of female reproductive organs [19].

CAP elicits a sensation of burning pain by selectively activating sensory neurons that convey information about noxious stimuli to the central nervous system. An expression cloning strategy was used based on calcium influx to isolate functional cDNA encoding a capsaicin receptor from sensory neurons. This receptor is a non-selective cation channel that is structurally related to members of the TRP family of ion channels called transient receptor potential vanilloid type-1 (TRPV1). In summary, TRPV1 is a channel activated by CAP. The effects of CAP are mediated through TRPV1 [20].

In order to gain more insight into the effect of CAP on spermatogenesis, we investigated the impact of this compound on germ cells by using previously developed rat spermatogonial stem cell lines [21] as a model. We studied herein the expression of TRPV1 on the germ cells and our results indicate that CAP induces apoptosis of the immortalized cell lines in a time and dose-dependent manner and that the effect may be mediated by TRPV1 which is expressed by these cells.

## Methods

### Animals and cell lines

Adult Wistar U: WU (CpB) male rats were obtained from the Central Animal Facilities of the University of Utrecht, The Netherlands. All animals were killed by CO_2 _inhalation. The testes were immersion-fixed in Bouin's solution, paraffin-embedded, sectioned and processed for immunohistochemistry. The ethical and animal care board of the University of Utrecht approved this study. Two rat spermatogenic stem cell lines (Gc-5spg and Gc-6spg) were used [21]. A rat glioma cell line (A10-85) was purchased from the European Collection of cell cultures (ECACC).

### Cell culture

Gc-5spg and Gc-6spg cells were grown in MEM (Life Technologies, Inc., Paisley, Scotland, UK) supplemented with single strength nonessential amino acids, 100 IU/ml-100 μg/ml penicillin-streptomycin, 40 μg/ml gentamycin, 15 mM HEPES, 250 ng/ml Fungizone (all from Life Technologies, Inc.), 0.12% sodium bicarbonate, 4 mM L-glutamine, 10 ng/ml platelet-derived growth factor-BB, 10 ng/ml recombinant human basic fibroblast growth factor, 10 ng/ml recombinant human LIF, 20 μM forskolin (all from Sigma, St. Louis, MO), 1 μM β-E2-17-cypionate (ICN Biomedicals B.V., Zoetermeer, The Netherlands) and 2.5% FCS at 32°C and 5% CO_2 _in a humidified atmosphere. The culture dishes were precoated with a thin layer of dried matrigel [22]. Culture medium was replaced twice a week. At confluence, cells were passaged following trypsinization with 0.25% trypsin-ethylene diamine tetra-acetic acid (EDTA) solution (Life Technologies).

To study the effect of CAP, cells were seeded at 25 × 10^3 ^per cm^2 ^in tissue culture chambers that had previously been coated with matrigel (BD Biosciences, CA, USA) as above. 25 cm^2 ^flasks were used to determine apoptosis by flow cytometry and 4 wells labtek glass slide chambers were used for immunocytochemistry. After incubation overnight in passaging medium at 32°C, cells were refreshed with medium containing either 0 (control), 150 uM, 200 uM or 250 uM CAP (> 95% pure, Sigma, St. Louis, MO, USA). Control cells were treated with the solvent only (DMSO) at a concentration equal to that in a 250 μM CAP solution or with 1 μM Staurosporine (Cat no #1055682, Roche, IN, USA) for 24 and 48 hours. Incubations were performed for 24 or 48 hours.

### Immuno-histo- and cytochemistry

#### Anti-activated caspase-3 antibody staining

Cultured cells were fixed with Methacarnoy solution for 10 minutes at room temperature. Fixed cells were rinsed with PBS and blocked with 5% goat serum in 0.2% Tween 20/PBS. The cells were permeabilized with 0.1% Triton X-100 for 5 minutes at room temperature and incubated with affinity-purified rabbit anti-human caspase-3 active (R&D Biosystems, MN, USA; 1:2500 in blocking solution) overnight at 4°C. A biotinilated goat anti-rabbit secondary antibody (BA-1000 Vector Labs, Burlingame, USA; dilution 1:200) was then incubated for 2 hours at room temperature. The ABC kit (Vector Labs, Burlingame, USA) was used according to the manufacturer's instructions. Antibody reactivity was then detected by aminoethylcarbazole staining (AEC, Sigma). The cells were then counterstained with Mayer's Haemaluin, mounted with Paramount and studied. To monitor the specificity of the staining rabbit serum was used in place of the primary antibody.

#### Anti TRPV1 antibody staining

All incubations with live cells were performed on ice and during one hour. Cells were consecutively incubated with goat anti human polyclonal anti-TRPV1 antibody (Santa Cruz, dilution 1:50) rabbit anti-goat biotinilated secondary antibody (BA-5000 Vector Labs, Burlingame, CA, USA; dilution 1/200) and streptavidin-PE (dilution: 1/200. Pharmingen, Becton Dickinson Co., San Jose, CA, USA). Finally slides were fixed with 100% Methanol at -20°C for 10 minutes, mounted with Fluorosave and screened with a confocal laser scanning microscope (Leica TCS-SP). Negative controls were incubated with a TRPV1 blocking peptide (Santa Cruz).

Bouin's fixed, paraffin embedded 5 um-thick rat testis sections were deparaffinized and boiled in a microwave oven (700 Watt) 3 × 10 min in sodium citrate buffer (0.1 mM, pH = 6) for antigen retrieval. All subsequent incubations were performed for 1 hour at room temperature. The slides were then blocked with 5% goat serum in 1% BSA/PBS and incubated with the rabbit anti human – VR1 antibody (Neuromics, Edina, MN, USA; 1:500 in 1% BSA/PBS). Biotinilated goat anti-rabbit secondary antibody (BA-1000, Vector Labs; 1:200 in 1% BSA/PBS) was then applied. The ABC kit was finally used according to the manufacturer's instructions. Antibody reactivity was finally detected by diaminobenzidine staining (DAB, Sigma, St. Louis, MO, USA). Sections were counterstained with hematoxylin, dehydrated, mounted with Pertex and studied. Goat serum was applied on control sections.

### SDS-PAGE and Western blotting

Protein lysates from the cell lines Gc-5spg and Gc-6spg and the control glioma cell line (A10-85) were prepared in RIPA buffer (PBS, 1% NP40, 0.5% sodium deoxycholate, 0.1% SDS) including 1 mM phenylmethylsulfonylfluoride. Of each sample, 50 μg were separated on a 12% SDS-polyacrylamide gel and blotted onto a polyvinylidene fluoride membrane (Millipore Corp., Bedford, MA, USA). Western blots were blocked using Blotto-A, containing 5% Protifar (Nutricia, Zoetermeer, The Netherlands) in Tris-buffered saline (10 mM Tris; 150 mM NaCl, pH 7.6), including 0.05% Tween-20. Rabbit polyclonal anti-VR1 antibody (Neuromics) was diluted 1:1000 in Blotto-A and incubated for 1 h at room temperature. Blots were washed with Tris-buffered saline with 0.05% Tween-20. After incubation with goat anti-rabbit-HRP (P-0260 Dako Cytomation, 1:5000 in Blotto-A) secondary antibody for 1 h, blots were incubated with the electrochemiluminescence kit (ECL, Amersham Pharmacia Biotech, Little Chalfont, UK) and exposed to an x-ray film (RX-omat, Kodak, Chalone/Saone, France).

### Flow cytometric analysis of apoptosis

Treated cells were trypsinized and centrifuged for 3 min at 1000 × g in an Eppendorf centrifuge and pellets were resuspended in PBS. Aliquots of 100 μl (i.e. ca. 1 million cells) samples were vortexed at full speed for 30 s and were placed on ice.

900 μl pre-cooled 70% ethanol in PBS was added carefully to the samples and mixed. The mixture was centrifuged for 3 min 1000 × *g *and the pellet was resuspended in 1 ml PBS and centrifuged once more. The pellet was resuspended 33% PBS and 67% extraction buffer (192 mM Na_2_HPO_4 _and 4 mM citric acid, pH 7.8), vortexed, kept at room temperature for 10 min and revortexed. The mixture was then centrifuged for 3 min 1000 × *g *and the pellet was resuspended in 1 ml PBS containing 50 μg/ml propidium iodide and 50 μg/ml RNase and incubated for 30 min in the dark. The mixture was filtered to remove clumped material through a 22 μm gauge filter (Millipore, Billerica, MA, USA) and analysed on a FACScalibur (Becton and Dickinson, San Jose, CA, USA). The linear intensity of PI fluorescence was detected in FL-3 (630 nm long pass emission wavelength filter) for non-aggregated nuclear events. As a positive control, cells were treated with 1 uM of staurosporine during 24 and 48 hours. Flow cytometric experiments were repeated four times.

### Data analysis

The data were analyzed using the Univariate Analysis of Variance where experiments were taken as block factor, time and dose as fixed factors and positive control (staurosporine treatment) as covariate. Post-hoc pair-wise comparisons were performed using the Bonferroni correction for multiple testing.

## Results

### Capsaicin induces apoptosis in germ cells

The addition of different concentrations of CAP to the spermatogonial stem cell lines induced morphological changes resembling apoptosis. These changes were observed in either cell line and at both 24 and 48 hours.

**Figure 1:**
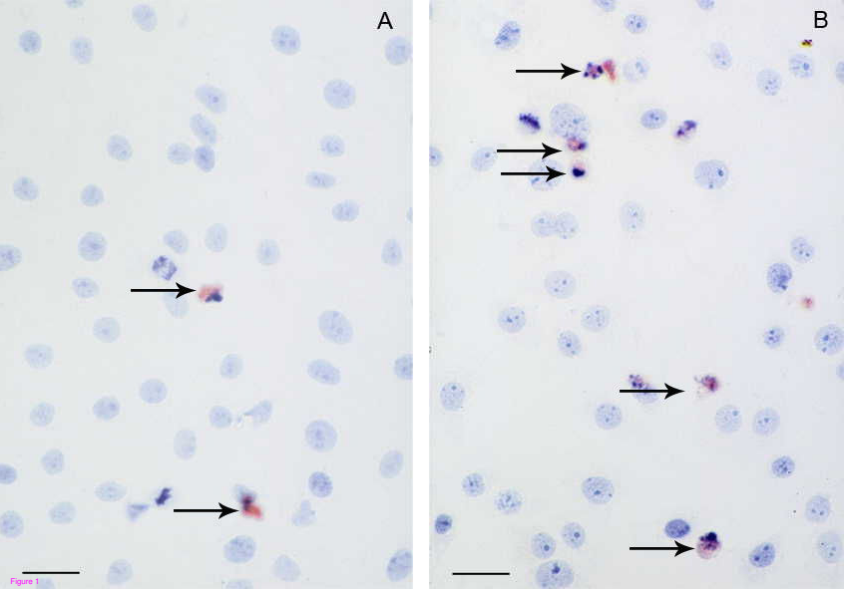
Photomicrograph of Gc-5spg stained with an anti-activated caspase 3 antibody and counterstained with Haemaluin as described in Materials and Methods. **A**, Control culture treated with DMSO at the same concentration as that used in the 250 uM CAP solution; **B**, Culture treated with 250 uM CAP during 48 h. Arrow shows apoptotic cells. Bars represent 15 um

To further investigate the occurrence of apoptosis, treated cultures were labeled for activated caspase 3. As shown in figures 1A and 1B, cells with altered morphology are increased in the CAP treated cultures compared to the control cultures and also expressed activated caspase 3, indicating that CAP caused apoptosis in the germ cells. This was further confirmed and quantified by studying CAP induced DNA fragmentation using flow cytometry and by determining hypodiploid DNA content stained with PI.

**Figure 2:**
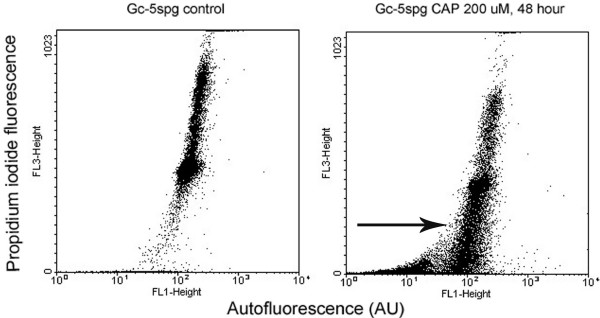
Flow cytometric determination of apoptotic germ cells. Representative dot blot histograms of Gc-5spg cells cultured in control medium (left panel) and in medium ontaining 200 μM CAP during 48 h (right panel). On the x-axis the FL1 fluorescence (autofluorescence) and on the y-axis the FL3 fluorescence (for PI) are indicated. The population of apoptotic cells (with hypodiploid DNA, arrow) becomes clear after incubation with CAP.

**Figure 3:**
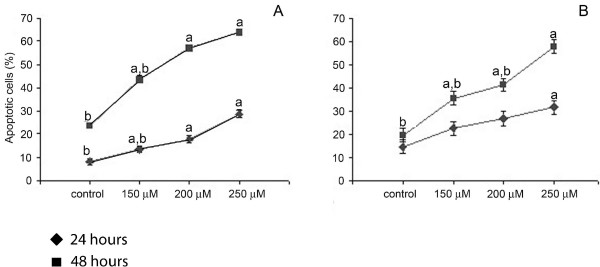
CAP triggers in a dose-dependent manner apoptosis of the Gc-5spg and Gc-6spg cell lines. Quantification of CAP induced apoptosis per time point and doses was based on data obtained from the flow cytometry analysis as illustrated in Fig. 2. **A**, Gc-5spg **B**, Gc-6spg. a represents statistically significant difference between control and the doses applied and b represents statistically significant difference between 24 h. of treatment and 48 h. of treatment.

As illustrated by the dot blot histograms (Fig. 2), CAP induced a significant shift in the number of apoptotic cells with hypodiploid DNA content in comparison to control cultures. The percentage of apoptotic cells from quadruplicate cultures was quantified and was found to significantly increase from about 8% and 14,6% respectively in the untreated Gc-5spg and Gc-6spg cell lines to 17,8% and 26,8% in respective cell lines with 200 μM CAP after 24 h. With increasing CAP concentrations, the effect was even more pronounced with both cell lines, and after either both 24 and 48 h (Fig. 3A and 3B). Staurosporine induced 52.3% and 56.2% underwent apoptosis after 24 and 48 hours respectively. Statistical analysis of the data demonstrated that the response of Gc-6spg was dependent on the incubation time, i.e. the shorter the incubation time the stronger the effect, while this was not the case for Gc-5spg. This might reflect intrinsic differences between these two cell lines [22].

### The transient receptor potential vanilloid receptor 1 (TRPV1) is expressed by premeiotic germ cells

Since CAP is a TRPV1 agonist and no information was available on the expression of this receptor in germ cells, we determined the expression of TRPV1 on the spermatogonial stem cells and also on germ cells *in vivo*.

**Figure 4:**
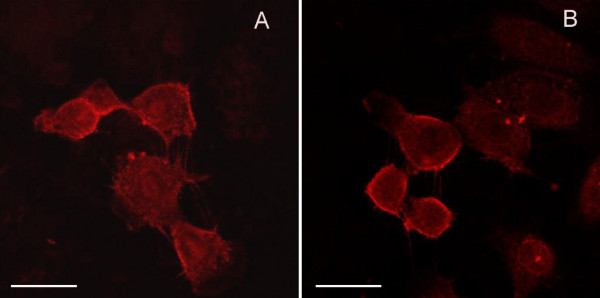
Images of Gc-5spg (A) and Gc-6spg (B) cells obtained after immunofluorescent labelling of the cells with an anti-TRPV1 antibody and CLS microscopy as described in Materials and Methods. Labelling was observed on the plasma membrane of the germ cells Bars: 20 um.

**Figure 5:**
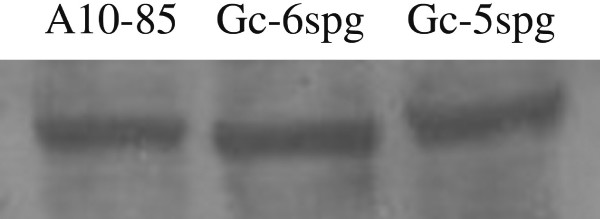
Detection of TRPV1 on Gc-5spg and Gc-6spg cell lines using Western Blotting. Lane 1, A10-85 Glioma cell line (positive control); lane 2, Gc-6spg; lane 3, Gc-5spg.

TRPV1 was localized on the Gc-5spg and Gc-6spg rat spermatogonial cell lines as determined by immuno-labeling and confocal microscopy. TRPV-1 reactivity was predominantly observed on the plasma membrane of both cell lines (Fig. 4A and 4B). The protein was also detected in both the positive control (protein extracts from a glioma cell line) and the Gc-5spg and Gc-6spg cell lines by a band migrating to 90 kD, the expected molecular weight (Fig. 5).

**Figure 6:**
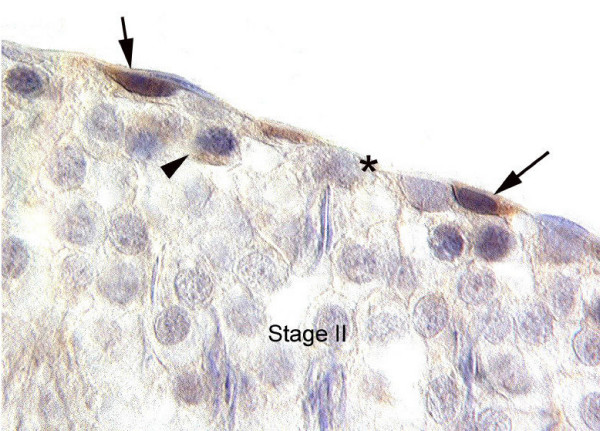
Photomicrograph of a section through an adult rat testis showing TRPV1 labelling of premeiotic germ cells, at stage II of the seminiferous epithelium. Arrow, undifferentiated spermatogonia; arrow head, early pachytene spermatocytes; asterisk, Sertoli cells. Bar represents 10 um.

TRPV1 was also expressed *in vivo *by premeiotic germ cells including both undifferentiated and differentiated spermatogonia independent of the stage of the epithelial cycle. Early spermatocytes only weakly expressed TRPV1 whereas no expression was detected in spermatids (Fig. 6).

## Discussion

Herein, we demonstrate that CAP can induce apoptosis in two different spermatogonial stem cell lines *in vitro*. In addition we show that the cell lines used and the germ cells from which the cell lines originated express the CAP receptor, TRPV1.

An increase in apoptosis following CAP treatment was demonstrated by using two independent methods, detection of activated caspase-3 by immuno-cytochemistry and quantification of DNA fragmentation by flow cytometry. Our observations are in accordance with previously reported findings and add to the list of cell types that respond to CAP by undergoing apoptosis. Interestingly, many of the pro-apoptotic effects of CAP have been observed with transformed cells and to a much lesser extent with their non-transformed/quiescent counterparts [10,11]. The spermatogonial cell lines used here are not transformed as they do not form tumors after in vivo transplantation [21]. However, metabolically, they are highly active as they continuously proliferate and this may underlie their CAP sensitivity.

The doses of CAP used in our cultures were based on previous reports in other cell types [22,23] and our experience with the spermatogonial stem cell lines. The effect of CAP on testes has previously been demonstrated by others, [24,25] but spermatogonia are located outside of the Sertoli cell blood barrier [26]. therefore we conducted our study with a direct exposure of spermatogonia to CAP. Although it is not very easy to extrapolate our findings to circulating levels of CAP when treating *in vivo*, our results are in line with the findings of *Nagabushan et al*. [27] who demonstrated a deleterious effect of CAP on the testis *in vivo*. These authors found a decrease in testicular DNA synthesis after CAP administration (by means of injection) to mice. The only proliferating cells in the adult testis are the spermatogonia which include the spermatogonial stem cells and the differentiating spermatogonia. Therefore the decrease in cell proliferation as described by these authors may only have been the result of a decrease in spermatogonial (stem cell) proliferation and/or apoptosis. Muralidhara et al. [17] did not observe any effect in vivo, perhaps due to the relatively low concentrations of CAP applied and the methods used to monitor testicular damage. Testicular weight and histology may not be sensitive enough to monitor changes in the spermatogonial germ cell compartment. The findings obtained with roosters [18] may be explained by the timing of CAP administration (juvenile *versus *adult animals), the length of exposure to CAP and by the difference in CAP sensitivity between mammals and birds [28]. TRPV1 is a cation channel which is activated by CAP [20], however this receptor is also sensitive to protons and temperatures above 43 oC [20]. TRPV1 excecuted effects could be regulated by ligands and regulatory mechanisms other then dietary CAP such as these.

The observation that CAP may be harmful to spermatogenesis may in turn be relevant within the context of testicular germ cell tumors (TGCT). These tumors arise from dysfunctional gonocytes, the so-called carcinoma in situ (CIS) cells [29] which remain quiescent during infancy and start proliferating at puberty to give rise to either seminoma, non seminoma or combined tumors [30]. It is known that TGCT are curable in most cases, yet effective therapies for advanced stages of the disease and for recurrent germ cells tumors still need to be developed [31]. As gonocytes resemble in many aspects the spermatogonial stem cells [32], and CIS and seminoma are very similar, our findings may suggest a potential use of CAP for the management of TGCT.

Many of the acute cellular effects associated with CAP occur via the interaction of CAP and TRPV1. Activation of this receptor leads to an increase of the intracellular [Ca^2+^] concentration which in turn can mediate apoptosis through various mechanisms [34]. TRPV1 is expressed by a number of neuronal and non-neuronal tissues. In particular TRPV1 mRNA has been detected in rat prostate, testis, penis and bladder tissue, and in all human genito-urinary tract tissues [34]. Recently, TRPV1 expression has also been demonstrated in cultured rat Sertoli cells [35]. We therefore set out to study the expression of this receptor in germ cells as this was not known. The spermatogonial stem cell lines as well as premeiotic germ cells in situ express TRPV1. Hence, CAP may affect germ cell survival through TRPV1. It is also possible though, that CAP induces apoptosis in the spermatogonial germ cell lines in a TRPV1 independent manner. Recently, we demonstrated that a lack of TRPV1 in TRPV1^-/- ^mice is deleterious to germ cell survival under heat stress conditions [36]. In other words, activation of TRPV1 by heat may induce factors that protect the germ cells from undergoing apoptosis instead of inducing apoptosis. Although the present and our previous study are not comparable as different models (mice *versus *rat, in vivo versus in vitro) and different TRPV1 agonists were used, it is indeed possible that CAP bypasses TRPV1 in the cultured cells. In fact, previous findings have indicated that concentrations of CAP in the range of 100 to 300 μM (as used here) and/or long-term exposure to this compound may interact with enzymatic processes either in the plasma membrane or in the mitochondria of cells that subsequently lead to cell death [8,37]. The cellular targets of CAP in the spermatogonial stem cell lines and the downstream effectors of germ cell apoptosis will be the focus of future research.

In contrast to the finding of Auzanneau et al[36] we did not observe TRPV1 expression in the Sertoli cells. This is possibly due to the difference in sensitivity of the methods used (i.e. cultured cells *versus *paraffin sections) and the use of different antibodies.

## Conclusion

In this study, we demonstrate that CAP induces apoptosis of mitotic germ cells *in vitro*, as evidenced by morphology, caspase activation and nuclear fragmentation. The germ cells used, express TRPV1. It remains to be investigated whether this receptor is involved in the CAP mediated apoptosis of the germ cells.

## Competing interests

The authors declare that they have no competing interests.

## Authors' contributions

SCM participated together with FMFvD-E in the design of the study. The experiments were carried out by SCM. Data analysis was performed by SCM, BMG, AMMvP, FMFvD-E. The manuscript was written by SCM. AMMvP, HE, AO, BMG and FMFvD-E critically read the manuscript. All authors have approved the final manuscript.
