# Correction: Spermatogonial stem cell sensitivity to capsaicin: An in vitro study

**DOI:** 10.1186/1477-7827-9-17

**Published:** 2011-01-28

**Authors:** Sefika C Mizrak, Bart M Gadella, Hatice Erdost, Aytekin Ozer, Ans MM van Pelt, Federica MF van Dissel-Emiliani

**Affiliations:** 1 Fertility Laboratory, Centre for Reproductive Medicine, Academic Medical Centre, Amsterdam, The Netherlands; 2 Department of Biochemistry and Cell Biology, Faculty of Veterinary Medicine, Utrecht University, Utrecht, The Netherlands; 3 Histology and Embryology Department, Faculty of Veterinary Medicine, Uludag University, Bursa, Turkey; 4 Department of Equine Sciences, Faculty of Veterinary Medicine, Utrecht University, Utrecht, The Netherlands

## 

Since publication of our article [1], we have realised that the legend of Figure one needs to be corrected and should read as follows:

"Photomicrograph of Gc-5spg and Gc-6spg stained with an anti-activated caspase 3 antibody and counterstained with Haemaluin as described in Materials and Methods. A, Gc-5spg culture treated with 200 uM CAP for 24 h B, Gc-6spg culture treated with 250 uM CAP during 48 h."

We apologise for any confusion this may have caused.
